# Breast cancer, cytomegalovirus and Epstein–Barr virus: a nested case–control study

**DOI:** 10.1038/sj.bjc.6605675

**Published:** 2010-04-20

**Authors:** B Cox, A Richardson, P Graham, R E Gislefoss, E Jellum, H Rollag

**Affiliations:** 1 Department of Preventive and Social Medicine, Hugh Adam Cancer Epidemiology Unit, Dunedin School of Medicine, University of Otago, Dunedin, New Zealand; 2 Community and Public Health, Canterbury District Health Board, Christchurch, New Zealand; 3 Department of Public Health and General Practice, University of Otago, Christchurch, New Zealand; 4 Cancer Registry of Norway, Institute of Population-based Cancer Research, Oslo, Norway; 5 Institute of Clinical Biochemistry, Faculty of Medicine, University of Oslo, Rikshospitalet, Oslo, Norway; 6 Institute of Microbiology, Faculty of Medicine, University of Oslo, Rikshospitalet, Oslo, Norway

**Keywords:** breast cancer, cytomegalovirus, Epstein–Barr virus

## Abstract

**Background:**

We investigated whether elevation in serum cytomegalovirus (CMV) or Epstein–Barr virus (EBV) immunoglobulin G (IgG) antibody levels precedes the development of breast cancer.

**Methods:**

A nested case–control study was carried out within the Janus Serum Bank cohort. Two serum samples, one taken at least 4 years before diagnosis (sample 2) and an earlier sample (sample 1) from 399 women with invasive breast cancer and from 399 controls, matched for date of blood samples and age were tested for CMV and EBV IgG antibodies. Odds ratios (ORs) with 95% confidence intervals (CIs) for CMV and EBV seroconversion between the samples and unit changes in IgG optical density (OD) examined as a continuous variable were calculated using conditional logistic regression.

**Results:**

Eleven cases and three controls seroconverted for CMV IgG between the first and second blood samples, with an adjusted OR for CMV IgG seroconversion of 4.0 (95% CI=1.1–14.4). The risk of breast cancer, adjusted for parity, increased per unit difference in CMV OD between samples (OR=1.7, 95% CI=1.1–2.5). In an analysis restricted to parous cases and age-matched parous controls, the OR for CMV seroconversion for IgG between the two samples, adjusted for parity and age at first birth, was 9.7 (95% CI=1.2–77.3). The EBV seroconversion or change in EBV OD was not associated with risk of breast cancer.

**Conclusion:**

Our hypothesis that elevation in serum CMV IgG antibody levels precedes the development of breast cancer in some women is supported by the results of this study. Changes in EBV IgG antibody are not associated with risk of breast cancer.

There are major geographical differences in the age-standardised incidence of breast cancer (Ferlay *et al*, 2004) that cannot be entirely explained by variations in known risk factors between countries. Although differences in reproductive factors among populations are important, a similar geographical pattern for breast cancer in men has been found (Thomas, 1993).

Mouse mammary tumour-like viruses and Epstein–Barr virus (EBV) have been suggested to cause breast cancer, but the evidence that either is associated with breast cancer has been inconsistent (Xue *et al*, 2003; Glaser *et al*, 2004; Mant and Cason, 2004; Szabo *et al*, 2005). Recently, cytomegalovirus (CMV) has been linked to the development of inflammatory diseases and cancer (Söderberg-Naucler, 2006).

It has been hypothesised that breast cancer can be caused by late exposure to a common virus (Richardson, 1997). Breast cancer incidence is frequently higher in countries where exposure to CMV may occur late than in countries where almost everyone is exposed in childhood. A strong negative inter-country correlation (Pearson's correlation coefficient −0.79, *P*<0.0001) was found between breast cancer incidence and the percentage of adults who are CMV seropositive (Richardson, 1997).

Delayed exposure to EBV (or CMV), measured by illness from infectious mononucleosis, has been suggested to increase risk of breast cancer (Yasui *et al*, 2001), but recall bias may have influenced the results.

In an Australian case–control study, cases and controls did not differ in seropositivity for CMV or EBV (Richardson *et al*, 2004). However, in seropositive women, mean immunoglobulin G (IgG) values were higher in cases than controls for CMV (1.20 *vs* 0.98 optical density (OD), *P*=0.005), but not for EBV (2.65 *vs* 2.57 OD, *P*=0.5). The adjusted odds ratios (ORs) per OD unit were 1.46 (95% confidence interval (CI)=1.06–2.03) for CMV IgG and 1.11 (95% CI=0.93–1.33) for EBV IgG. We hypothesised that the higher mean IgG levels found in women with breast cancer could be the result of more recent infection with CMV, and may indicate that late exposure to CMV (in adulthood rather than childhood) is a risk factor for breast cancer. Limitations of this work were that it was retrospective, with blood samples collected after the diagnosis of breast cancer, and only women aged <40 years were studied.

## Materials and Methods

To investigate whether CMV IgG levels were increased before the diagnosis of breast cancer, a case–control study nested in the cohort of female donors to the Janus Serum Bank in Norway was undertaken. The Janus project was started in 1973 to collect and store blood samples from healthy people for later scientific use. Participants were recruited from several counties in Norway during routine health examinations or in conjunction with screening for risk factors of cardiovascular diseases. The participation rate was 85% during 1974 to 1978 and 75% during 1986 to 1991. Samples were also collected from blood donors from the Red Cross Blood Donor Centre in Oslo. The serum bank contains samples from approximately 333 000 people (151 000 women) and 10% are blood donors. The sera have been stored at −25°C (Jellum *et al*, 1993, 1995).

The stored blood samples from cases and controls were tested for CMV and EBV IgG antibodies. The CMV and EBV antibody levels in stored blood remain stable despite prolonged storage (Jellum *et al*, 1993; Pappin *et al*, 1995; Levin *et al*, 2003). The study was approved by the Regional Ethics Committee of Southern Norway and Data Inspectorate, Norway.

### Selection of cases and controls

Cases were randomly selected from women in the Janus Serum Bank cohort with invasive breast cancer who had been identified by linkage to the Norwegian Cancer Registry until 400 cases were attained. Women were eligible to be cases if they were aged 20 years and over at diagnosis, with a blood sample taken 4 or more years before the diagnosis of breast cancer (the index sample), and a blood sample at least 12 months earlier than the index sample. Eligible controls were women from the cohort who were alive and free of cancer (other than squamous or basal cell carcinoma of the skin) at the time that the case was diagnosed. They were frequency matched to the cases by 5-year age group and had a blood sample taken within ±2 months of the index sample of the case. From these eligible controls, women with at least one earlier sample were randomly selected. These controls were individually matched to cases by duration of sample storage (±2 months) of the earlier sample. Where it was not possible to find a control in which the earlier sample could be matched for duration of storage, an eligible control with the closest available early sample was randomly selected. The two samples for each case and individually matched control had to be separated in time by at least 12 months to allow adequate time for IgG levels to change between the samples, as CMV and EBV IgG titres rise initially after infection and then gradually decline, with residual antibody detectable for several years (IARC Working Group on the Evaluation of Carcinogenic Risks to Humans, 1997; Mendez *et al*, 1999). Table 1 shows the selection criteria for cases and controls.

### Sample size calculations

On the basis of our earlier results (Richardson *et al*, 2004), samples from 400 cases and 400 controls were predicted to provide, for the assessment of CMV and EBV IgG levels, at least 80% power to detect a difference of 0.15 units of OD in mean CMV IgG levels between cases and controls (a difference of 21.4% of the s.d.), with *α*=0.05. As we also wished to examine changes in CMV IgG and EBV IgG levels over time in cases and controls, two samples for each case and each control were tested. We had no estimates of the magnitude of changes in CMV or EBV IgG over time or the rate of seroconversion, so they were not used to estimate the sample size or statistical power of the study, however, the CIs indicate the precision of the ORs obtained. In total, 1600 samples were tested; 800 from the cases (two samples from each of the 400 cases) and 800 from the controls (two samples from each of the 400 controls).

Conditional logistic regression using COXREG in SPSS (SPSS Inc., 2007) was used to estimate ORs for factors associated with the risk of breast cancer. The date of diagnosis was taken as the reference date for each case and their matched control for the calculation of age. Parity and age at first child were obtained from the information recorded when the second blood sample was drawn and were included in the analysis. Odds ratios, with 95% CIs, for CMV and EBV unit changes in IgG OD, and differences in values between samples, examined as continuous variables and adjusted for parity, as well as CMV and EBV seroconversion, were calculated. For analysis in parous women, adjustment for parity and age at first child was also undertaken. Analyses for women diagnosed before 1998 and from 1998 onwards and their matched controls were undertaken to assess whether the introduction of the national breast screening programme in Norway in 1998 influenced the results. For 572 subjects, height and weight were measured as part of the assessment of cardiovascular risk factors within the cohort resulting in 277 case–control pairs with body mass index (BMI) measurements for analysis (69% of all case–control pairs). Body mass index was categorised into four groups: <22, 22.1–24, 24.1–26 and >26. Use of hormone replacement therapy was not recorded.

### Serological tests

The serological tests were carried out in Norway at the Institute of Microbiology, Rikshospitalet, Oslo, Norway. Each blood sample was tested using standard enzyme immunoassays for CMV IgG and EBV viral capsid antigen IgG. The serum specimens were organised as 32 batches, each comprising 12 sets. Two specimens from a case and two specimens from the corresponding control comprised one set. The specimens within a batch were then mixed. Specimens from the same set were in the same batch, but not necessarily next to each other.

The CMV IgG and EBV-VCA antibody internal control sera were placed in wells in the first row and last row of each test plate. If the OD of these two wells on the test plate differed by >20%, the results were rejected and the sera reanalysed. In addition, an internal laboratory control was analysed on each plate to control inter-assay variations.

The CMV IgG antibodies were assessed by Enzygnost Anti-CMV/IgG test kit, Dade Behring, Marburg, Germany. The OD cutoff value was 0.200. A reference serum provided with the kit was used as a correction factor to standardise the OD value between test plates. The EBV-VCA IgG antibodies were assessed by Novitec EBV-VCA IgG test kit, Hiss Diagnostics, Freiburg, Germany. The OD cutoff value for this test was also 0.200.

## Results

Cases of breast cancer were diagnosed between February 1982 and December 2003, with 53% of cases diagnosed from 1998 onwards. One woman with DCIS only and her matched control were excluded, leaving 399 case–control pairs for analysis. The distribution of age and parity in cases and controls is shown in Table 2. The age distribution of cases and controls was similar due to matching on age. Parity was significantly different between cases and controls (χ^2^=12.83, 5 df, *P*=0.025) with similar proportions of nulliparous women, but fewer women with four or more live births among cases.

For the CMV IgG antibodies, the positive control provided with the kit had a mean OD value of 0.825±0.110 and the internal laboratory standard had a mean OD value of 0.235±0.052. For the EBV-VCA IgG antibodies, the positive control provided with the kit had a mean OD value of 2.610±0.230 and the internal laboratory standard had a mean OD value of 0.900±0.13.

The average time between serum samples was 8.5 years (range 1–17 years). Of the cases, 314 (78.7%) were seropositive for CMV at their second serum sample compared with 329 (82.5%) of the controls. Eleven cases and three of the controls seroconverted for CMV IgG between the first and second samples (*P*=0.03). This effect was largely confined to parous women (10 cases and 2 controls). The mean CMV IgG for cases for sample 1 was 1.09 OD (s.d.=0.77) and the mean CMV IgG for controls was 1.18 OD (s.d.=0.71). The mean CMV IgG for cases for sample 2 was 1.19 OD (s.d.=0.79) and the mean CMV IgG for controls was 1.22 OD (s.d.=0.72). No significant differences in CMV IgG, with adjustment for parity, were found between cases and controls for sample 1 or 2 (ORs 0.84, 95% CI=0.69–1.03 and 0.95, 95% CI=0.78–1.15, respectively).

The mean EBV IgG for cases for sample 1 was 2.08 OD (s.d.=0.80) and the mean EBV IgG for controls was 2.04 OD (s.d.=0.73). The mean EBV IgG for cases for sample 2 was 2.19 OD (s.d.=0.82) and the mean EBV IgG for controls was 2.17 OD (s.d.=0.80). With adjustment for parity, no significant differences in EBV IgG were found between cases and controls for sample 1 or 2 (ORs 1.04, 95% CI=0.86–1.27 and 1.03, 95% CI=0.85–1.23, respectively). For EBV, 386 (96.7%) of cases were seropositive when the second sample was taken compared with 384 (96.2%) of the controls. Two cases and three controls seroconverted for EBV IgG between the first and second samples (Table 3).

The CMV IgG levels did not appear to be affected by the duration of storage of the serum samples and no significant correlations between CMV IgG or EBV IgG level and duration of storage were found. There was no difference in the overall distribution of CMV IgG and EBV IgG levels in cases compared with controls.

Table 4 shows the risk of breast cancer by parity, age at first child, BMI and seroconversion for CMV and EBV. There were 343 cases and 344 controls with at least one birth. Among the 301 case–control pairs who were both parous, there was no statistically significant difference in age at first birth (χ^2^=8.11, 4 df, *P*=0.09). Although a significantly increased risk of breast cancer was observed for parous women whose first birth occurred at age 28 years or older, no increased risk with increasing age at first birth with adjustment for parity was observed (OR=1.02, 95% CI=0.98–1.06). Nulliparity did not confer reduced risk (OR=1.0, 95% CI=0.6–1.5). The risk of breast cancer was significantly lower for women with five or more children compared with nulliparous women (OR=0.3, 95% CI=0.2–0.7), with or without adjustment for age at first birth or CMV IgG seroconversion, and was similar with additional adjustment for BMI for the 277 case–control pairs for whom it was possible. In parous women, with adjustment for age at first birth, the risk of breast cancer decreased with increasing parity with an OR of 0.8 (95% CI=0.7–0.9) per additional child after the first and was not appreciably changed with additional adjustment for age at first birth.

Seroconversion for CMV IgG between the first and second samples occurred for 11 cases and 3 controls, producing an unadjusted OR of 3.7 (95% CI=1.0–13.1). When adjusted for parity, the OR for CMV IgG seroconversion was 4.0 (95% CI=1.1–14.4). This was unaltered by adjustment for the length of time between tests, and was similar when restricted to case–control pairs for diagnoses before 1998 or 1998 onwards, OR=4.3 (95% CI=0.5–38.4) and OR=3.9 (95% CI=0.8–18.9), respectively. When only case–control pairs who were parous were included in the analysis (10 cases and 2 controls seroconverted), the unadjusted OR for CMV IgG seroconversion was 9.0 (95% CI=1.1–71.0), and this was increased slightly to 9.7 (95% CI=1.2–77.3) when adjusted for parity and age at first birth. The estimate was not altered significantly when the analysis was restricted to women over 50 years of age at diagnosis or reference date; OR=9.9 (95% CI=1.2–78.8).

When the analysis was restricted to the subgroup for whom BMI was measured, the OR for the association between CMV seroconversion and breast cancer with adjustment for parity was 3.7 (95% CI=0.7–18.5) and 3.7 (95% CI=0.7–18.4) when adjusted for parity and BMI. No trend in the ORs over the four categories of BMI, with adjustment for parity, was observed (OR=0.99, 95% CI=0.86–1.15). When restricted to parous women in this subgroup, the OR for CMV seroconversion was 7.6 (95% CI=0.9–64) and was unchanged with additional adjustment for age at first birth.

Changes in CMV serology from positive to negative, or staying positive or staying negative, between samples were not associated with risk of breast cancer. Only 2 cases and 3 controls seroconverted for EBV IgG between the two samples.

The crude OR per unit of difference in CMV OD between samples was 1.6 (95% CI=1.0–2.3), and the crude OR per unit difference in EBV OD between samples was 0.9 (95% CI=0.7–1.3). Table 5 gives ORs for seroconversion and unit changes in IgG values between samples, adjusted for parity and, for parous women, with additional adjustment for age at first birth. The OR per unit difference in CMV OD between samples, adjusted for parity, was 1.7 (95% CI=1.1–2.5), which was reduced to 1.6 (95% CI=1.0–2.5) when those that seroconverted for CMV IgG were excluded. The OR per unit difference in EBV OD between samples, adjusted for parity, was 1.0 (95% CI=0.7–1.3). In parous women, the ORs per unit difference in CMV OD and EBV OD, adjusted for parity and age at first child, were 2.0 (95% CI=1.2–3.4) and 1.0 (95% CI=0.7–1.4), respectively.

## Discussion

Our hypothesis that elevation in serum CMV or EBV IgG antibody levels precedes the development of breast cancer in some women is supported by the results of this study. Seroconversion of CMV IgG and increasing IgG levels between the samples were both associated with an increased risk of breast cancer among parous women.

The Janus serum bank cohort was ideal for this study because it meant that blood, which was collected years before the diagnosis of breast cancer, could be tested, so CMV and EBV IgG levels for cases are unlikely to have been affected by breast cancer and were not affected by treatment. Exposure to CMV and EBV was determined objectively using standard enzyme immunoassays for CMV IgG and EBV viral capsid antigen IgG, and laboratory staff were blinded to the case or control status of the samples, so these results are not vulnerable to recall bias or observer bias. The similar risk for case–control pairs before and after 1998, the year the Norwegian Breast Cancer Screening Programme began, suggests that the results were not affected by detection bias associated with screening.

Adjustment for the length of time between samples did not alter the results obtained and suggests that recent seroconversion rather than the duration of seropositivity may be the more important determinant of risk of breast cancer. Whether CMV remains in breast tissue after infection in human beings is not known. Increased CMV IgG levels may occur from recrudescence of latent infection or re-exposure to CMV without clinical infection. Increasing risk of breast cancer with increasing CMV OD might also occur if a significant risk factor for breast cancer was closely associated with recurrent CMV exposure.

Day care workers and mothers are at increased risk of acquiring CMV from children they care for (Bright and Calabro, 1999; Joseph *et al*, 2005; Noyola *et al*, 2005), and this combined with the recognised increased risk of breast cancer within 10 years of a full-term birth (Lambe *et al*, 1994) might explain an association in parous women. However, when we restricted our analysis to women who were 50 or more years of age at diagnosis or reference date in the controls, the strong effect of recent adult CMV infection in parous women remained, so this is an unlikely explanation of our results. The specificity of the association (for CMV, but not for EBV) supports a causal association between adult CMV infection and breast cancer in some parous women because if the results were due to bias or confounding an association between breast cancer and both CMV and EBV infection would be more likely than either by itself. Although the results for the women with measurements of BMI suggest that BMI is not likely to be a confounder of the associations found between changes in CMV IgG and breast cancer, other potential confounders, such as the use of hormone replacement therapy or alcohol consumption, were not able to be included in the analysis. Further investigation of a possible function of CMV infection in the presentation of breast cancer is needed.

The risk of adult CMV infection might be expected to increase with increasing parity, but increasing parity is associated with a reducing risk of breast cancer. However, if the prevalence of pre-invasive disease increased with age and exposure to CMV only promoted the progression of pre-invasive disease, then infection from young children after an early age of first full-term birth or increasing parity would provide some immunity against infection at an older age, after the onset of pre-invasive disease, and thus reduce the risk of breast cancer. Then both early age of first birth and increasing parity would, as has been observed, reduce the lifetime risk of breast cancer. Moreover, the increased risk of breast cancer observed within 10 years of first full-term birth (Lambe *et al*, 1994) could be mediated through an increased chance of exposure to CMV after first full-term birth. However, this hypothesis does not explain the lack of an increased risk of breast cancer in the 10-year period after first full-term birth for women whose first full-term birth occurs when 35 or more years of age (Lambe *et al*, 1994). The observed increase in risk of breast cancer in parous women with CMV IgG seroconversion between samples was present for women diagnosed at 50 or more years of age, suggesting that CMV infection after 35 years of age may also increase risk of breast cancer. The increased risk observed was present for few women in this study, suggesting that CMV infection may only be involved in the development of a minority of breast cancers.

Studies involving the long-term follow-up of women who have been, and not been, infected with CMV are required to verify the findings of this study. A greater time period between blood samples would be expected to increase the proportion of women for whom CMV IgG seroconversion occurs and provide more definitive examination of the association with breast cancer and whether risk increases with increasing age of adult CMV infection. Assessment of whether the increased risk of breast cancer in the 10-year period after first full-term birth varies by occurrence of CMV infection during the period is also needed.

## Figures and Tables

**Table 1:** Eligibility of cases and controls

**Cases**	**Controls**
Women aged 20 years and over at diagnosis with invasive breast cancer (identified by linkage to the Norwegian Cancer Registry)	Women alive without cancer (other than basal or squamous cell skin cancer) at the time the case was diagnosed, aged in the same 5-year age group as the case
Available stored blood sample taken at least 4 years before the diagnosis of breast cancer (the ‘index sample’)	Available stored blood sample taken within ±2 months of the case's index sample
Earliest available stored blood sample (taken at least 1 year earlier than the index sample described above)	Available early sample (preferably taken within ±2 months of the case's sample, but otherwise the closest early sample taken at least 1 year earlier than the sample described above)

**Table 2:** Characteristics of cases and controls

**Characteristic**	**Number of cases (%)**	**Number of controls (%)**
*Age (years)*		
20–44	12 (3.0%)	11 (2.8%)
45–49	16 (4.0%)	19 (4.8%)
50–54	52 (13.0%)	58 (14.5%)
55–59	87 (21.8%)	79 (19.8%)
60–64	91 (22.8%)	100 (25.1%)
65–69	93 (23.3%)	83 (20.8%)
70+	48 (12.0%)	49 (12.3%)
		
*Number of children*		
None	56 (14.0%)	55 (13.8%)
1	55 (13.8%)	45 (11.3%)
2	132 (33.1%)	124 (31.1%)
3	103 (25.8%)	92 (23.1%)
4	39 (9.8%)	46 (11.5%)
5+	14 (3.5%)	37 (9.3%)
		
*Age at first child (for parous women only) (years)* a		
0–20	73 (21.3%)	78 (22.7%)
21–22	72 (21.0%)	64 (18.6%)
23–24	55 (16.0%)	73 (21.2%)
25–27	68 (19.8%)	73 (21.1%)
28+	75 (21.9%)	56 (16.3%)
		
*BMI* b		
⩽22	88 (30.4%)	82 (29.0%)
21.1–24	70 (24.2%)	64 (22.6%)
24.1–26	49 (17.0%)	59 (20.8%)
>26	82 (28.4%)	78 (27.6%)

Abbreviation: BMI=body mass index.

a Restricted to 343 cases and 344 controls with at least one live birth.

b Restricted to 289 cases and 283 controls with measurements of BMI.

**Table 3:** CMV and EBV IgG results for cases and controls

	**Cases sample 2**		**Control sample 2**	
**Sample 1**	**Negative**	**Positive**	**Negative**	**Positive**
*CMV*				
Negative	79	11	65	3
Positive	6	303	5	326
				
*EBV*				
Negative	5	2	5	3
Positive	8	384	10	381

Abbreviations: CMV=cytomegalovirus; EBV=Epstein–Barr virus; IgG=immunoglobulin G.

**Table 4:** Unadjusted odds ratios for breast cancer by parity, age at first birth, CMV IgG and EBV IgG seroconversion

**Characteristic**	**Number of cases**	**Number of controls**	**Odds ratio for breast cancer**	**(95% CI)**
*Nulliparity*				
No	343	344	1.0	
Yes	56	55	1.0	(0.6–1.5)
				
*Number of children*				
None	56	55	1.0	
1	55	45	1.1	(0.7–2.0)
2	132	124	1.1	(0.7–1.7)
3	103	92	1.0	(0.6–1.7)
4	39	46	0.8	(0.4–1.5)
5+	14	37	0.3	(0.2–0.7)
				
*Age at first child (for parous women only) (years)* a				
0–20	63	72	1.0	
21–22	68	57	1.4	(0.9–2.4)
23–24	47	66	0.9	(0.5–1.5)
25–27	59	61	1.2	(0.7–2.0)
28+	64	45	1.8	(1.0–3.3)
				
*BMI* b				
⩽22	83	81	1.0	
21.1–24	67	63	1.1	(0.6–1.7)
24.1–26	46	57	0.8	(0.4–1.3)
>26	81	76	1.0	(0.6–1.6)
				
*Seroconversion for CMV IgG between samples*				
No	388	396	1.0	
Yes	11	3	3.7	(1.0–13.0)
				
*Seroconversion for CMV IgG between samples in parous women* a				
No	333	342	1.0	
Yes	10	2	9.0	(1.1–71.0)
				
*Seroconversion for EBV IgG between samples*				
No	397	396	1.0	
Yes	2	3	0.7	(0.1–4.0)

Abbreviations: BMI=body mass index; CI=confidence interval; CMV=cytomegalovirus; EBV=Epstein–Barr virus; IgG=immunoglobulin G.

a Restricted to 301 cases and 301-matched controls with at least one live birth.

b Restricted to 277 cases and 277-matched controls with BMI available.

**Table 5:** Adjusted^a^ odds ratios (95% confidence intervals) for CMV and EBV seroconversion and unit changes in IgG OD

	**All women**		**Parous women**	
	**Odds ratio**	**95% CI**	**Odds ratio**	**95% CI**
CMV IgG seroconversion	4.0	1.1–14.4	9.7	1.2–77.3
EBV IgG seroconversion	0.7	0.1–4.0	0.6	0.1–6.8
Unit change in CMV OD	1.7	1.1–2.5	2.0	1.2–3.4
Unit change in EBV OD	1.0	0.7–1.3	1.0	0.7–1.4

Abbreviations: CI=confidence interval; CMV=cytomegalovirus; EBV=Epstein–Barr virus; IgG=immunoglobulin G; OD=optical density.

a Results for all women are adjusted for number of births; results for 301 parous cases and their 301-matched parous controls are adjusted for number of births and age at birth of first child.
